# Supplementary material for: Heat adaptation of phage T7 under an extended genetic code
Source: Virus Evol. 2021 Dec 1;7(2):veab100. doi: 10.1093/ve/veab100 (PMC8923235; doi:10.1093/ve/veab100)
Supplement: veab100_Supp [file veab100_supp.zip › Sequencing_Analysis.zip!/Sequencing_Analysis/A1/evidence/RA_18.html]

BRESEQ :: Evidence


| Read alignment evidence... | | | | | | | | | | | |
| --- | --- | --- | --- | --- | --- | --- | --- | --- | --- | --- | --- |
|  | seq id | position | | ref | new | freq | score (cons/poly) | reads | annotation | genes | product |
| \* | NC\_001604.1 | 13,579 | 0 | C | T | 20.5% | 4406.9 / inf | 2320 | intergenic (–/–) | *–/–* | –/– |
| Reads supporting (aligned to +/- strand):  **ref** base C (854/963);  **new** base T (218/251);  **total** (1092/1228) | | | | | | | | | | | |
| Fisher's exact test for biased strand distribution *p*-value = 8.76e-01 | | | | | | | | | | | |
| Kolmogorov-Smirnov test that lower quality scores support variant *p*-value = 9.38e-01 | | | | | | | | | | | |

Only 100 of 2384 total aligned reads displayed.  

`ACTTTCCAGACGACTACGAGAAGTTTCGCTGGAGAGTCCCATTCTAATACGACTCACTAAAGGAGACACACCA‑TGTTCAAACTGATTAAGAAGTTAGGCCAACTGCTGGTTCGTATGTACAACGTGGAAGCCAAGCGACTGAACGATGAGGCTCGTAAAGAGGCCACACAGTCACGCGCTCTGGCGATTCGCTCCAACGAACTGGCTGACAGTGCATCCACTAAAGTTACCGAGGCTGCCCGTGTGGCAAACCAAGCTCAACAGCTTTCCAAATTCTTTGAGTAATCAAACA‑GGAGAAACCATTATGTCTAACGTAGCTGAAACTATCCGTCTATCCGATACAGCTGACCAGTGGAACCGTCGAGTCCACATCAACGTTCGCAACGGTAAGGCGACTATGGTTTACCGCTGGAAGGACTCTAAGTCCTCTAAGAATCACACTCAGCGTATGACGTTGACAGATGAGCAAGCACTGCGTCTGGTCAATGCGCTTACCAAAGCTGCCGTGACAGCAATTCATGAAGCTGGTCGCGTCAATGAAGCTATGGCTATCCTCGACAAGATTGATAACTAAGAGTGGTATCCTCAAGGTCGCCAAAG  >  NC_001604.1/13280‑13879`  

                                                                                                                                                                                                                                                                                                             `|`                                                                                                                                                                                                                                                                                                              
 `cTTTCCAGACGACTACGAGAAGTTTCGCTGGAGAGTCCCATTCTAATACGACTCACTAAAGGAGACACACCA‑TGTTCAAACTGATTAAGAAGTTAGGCCAACTGCTGGTTCGTATGTACAACGTGGAAGCCAAGCGACTGAACGATGAGGCTCGTAAAGAGGCCACACAGTCACGAGCTCTGGCGATTCGCTCCAACGAACTGGCTGACAGTGCATCCACTAAAGTTACCGAGGCTACCCGTTGTGCAAACCACGCTCAACAGCTTACAAACTTCTTTTAGTCATCAAAAC‑GGAAAAACCa                                                                                                                                                                                                                                                                                                            >  1:499410/1‑301 (MQ=255)`  
     `ccAGACGACTACGAGAAGTTTCGCTGGAGAGTCCCATTCTAATACGACTCACTAAAGGAGACACACCA‑TGTTCAAACTGATTAAGAAGTTAGGCCAACTGCTGGTTCGTATGTACAACGTGGAAGCCAAGCGACTGAACGATGAGGCTCGTAAAGAGGCCACACAGTCACGCGCTCTGGCGATTCGCTCCAACGAACTGGCTGACAGTGCATCCACTAAAGTTACCGAGGCTGCCCGTGTGGCAAACCAAGCTCAACAGCTTTCCAAATTCTTTGAGTAATCAAACA‑GGAGAAATCAATAT                                                                                                                                                                                                                                                                                                        <  1:444402‑M1/301‑1 (MQ=255)`  
     `ccAGACGACTACGAGAAGTTTCGCTGGAGAGTCCCATTCTAATACGACTCACTAAAGGAGACACACCA‑TGTTCAAACTGATTAAGAAGTTAGGCCAACTGCTGGTTCGTATGTACAACGTGGAAGCCAAGCGACTGAACGATGAGGCTCGTAAAGAGGCCACACAGTCACGCGCTCTGGCGATTCGCTCCAACGAACTGGCTGACAGTGCATCCACTAAAGTTACCGAGGCTGCCCGTGTGGCAAACCAAGCTCAACAGCTTTCCAAATTCTTTGAGTAATCAAACA‑GGAGAAACCATTAT                                                                                                                                                                                                                                                                                                        <  1:448470‑M1/301‑1 (MQ=255)`  
     `ccAGACGACTACGAGAAGTTTCGCTGGAGAGTCCCATTCTAATACGACTCACTAAAGGAGACACACCA‑TGTGCAAACTGATTAAGAAGTTAGGCCAACTGCTGGTTCGTATGTACAACGTGGAAGCCAAGCGACTGAACGATGAGGCTCGTAAAGAGGCCACACAGTCACGCGCTCTGGCGATTCGCTCCAACGAACTGGCTGACAGTGCATCCACTAAAGTTACCGAGGCTGCCCGTGTGGCAAACCAAGCTCAACAGCTTTCCAAATTCTTTGAGTAATCAAACA‑GGAGAAACCATTAT                                                                                                                                                                                                                                                                                                        <  1:360204‑M1/301‑1 (MQ=255)`  
                  `agaAGTTTCGCTTGAGAGTCCCATTCTAATACGACTCACTAAAGGAGACACACCA‑TGTTCAAACTGATTAAGAAGTTAGGACAACTGCTGGTTCGTATGTACAACGTGGAAGCCAAGCGACTGAACGATGAGGCTCGTAAAGAGGCCACACAGTCACGCGCTCTGGCGATTCGCTCCAACGAACTGGCTGACAGTGCATCCACTAAAGTTACCGAGGCTGCCCGTGTGGCAAACCAAGCTCAACAGCTTTCCAAATTCTTTGAGTAATCAAACA‑GGAGAAACCATTATGactgtctcttat                                                                                                                                                                                                                                                                                           >  1:184923‑M1/1‑289 (MQ=255)`  
                         `tCGCTGGAGAGTCCCATTCTAATACGACTCACTAAAGGAGACACACCA‑TGTTCAAACTGATTAAGAAGTTAGGCCAACTGCTGGTTCGTATGTACAACGTGGAAGCCAAGCGACTGAACGATGAGGCTCGTAAAGAGGCTACACAGTCACGCGCTCTGGCGATTCGCTCCAACGAACTGGCTGACAGTGCATCCACTAAAGTTACCGAGGCTGCCCGTGTGGCAAACCAAGCTCAACAGCTTTCCAAATTCTTTGAGTAATCAAACA‑GTAGAAACCATTATGatcgtttctgacatcgaag                                                                                                                                                                                                                                                                                    >  1:133414‑M1/1‑282 (MQ=255)`  
                          `cgatggaGAGTCCCATTCGAAAACGACTCAATAAAGGAGAAACACCA‑TGTTCAAACTGATTAAGAAGTTAGGCCAACGGCTGGTTAGTATGTACAACGTGGAAGCCAAGCGACTGAACGATGAGGCTCGTAAAGATGCCACACAGTCACGCGCTCTGGCGATTCGCTCCAACGAACTGGCTGACAGTGCATCCACTAAAGTTACCGAGGCTGCCAGTGTGGCAAACCAAGCTCAACAGCTTTCCAAATTCTTTGAGTAATCAAACA‑GGAGAAACCATTATGatcgtttctgacatcgaagc                                                                                                                                                                                                                                                                                   <  1:290467‑M1/298‑21 (MQ=255)`  
                                 `gagTCCCATTCTAATACGACTCACTAAAGGAGACACACCA‑TGTTCAAACTGATTAAGAAGTTAGGCCAACTGCTGGTTCGTATGTACAACGTGGAAGCCAAGCGACTGAACGATGAGCCTCGTAAAGAGGCCACACAGTCACGCGCTCTGGCGATTCGCTCCAACGAACTGGCTGACAGTGCATCCACTAAAGTTACCGAGGCTGCCCGTGTGGCAAACCAAGCTCAACAGCTTTCCAAATTCTTTGAGTAATCAAACA‑GGAGAAACCATTATGatcgtttctgacatcgaagccctgtct                                                                                                                                                                                                                                                                            >  1:281919‑M1/1‑274 (MQ=255)`  
                                  `gagacagttctaatacGACTCACTAAAGGAGACACACCA‑TGTTCAAACTGATTAAGAAGTTAGGCCAACTGCTGGATCGTATGTACAACGTGGAAGCCAAGCGACTGAACGATGAGGCTCGTAAAGAGGCCACACAGTCACGCGCTCTGGCGATTCGCTCCAACGAACTGGCTTACAGTGCATCCACTAAAGTTACCGAGGCTGCCCGTGTGGCAAACCAAGCTCAACAGCTTTCCAAATTCTTTGAGTAATCAAACA‑GGAGAAACCATTATGatcgtttctgacatcgaagctaacgccc                                                                                                                                                                                                                                                                           <  1:392860‑M1/294‑29 (MQ=255)`  
                                   `gTCCCATTCTAATACGACTCACTAAAGTAGACACACCA‑TGTTCAAACTTATTACAAAGTTAGGCCAACTGCTGGTTCGTATGTACAACGTGGAAGCCAAGCGACTGAACGATGAGGCTCGTAAAGAGGCCACACAGTCACGCGCTCTGACGATTCGCTCCAACGACCTGGCTGACAGTGCATCCACTAAAGTTACCGAGGCTGCCCGTGTGGCAAACCAAGCTCAACAGCTTTCCAAATTCTTTGAGTAATCAAATC‑GGAGAAACCATTATGaccgtttctgacatcgaagctaacgccct                                                                                                                                                                                                                                                                          >  1:307439‑M1/1‑272 (MQ=255)`  
                                   `gTCCCATTCTAATACGACTCACTAAAGGAGACACACCA‑TGTTCAAACTGATTAAGAAGTTAGGCCAACTGCTGGTTCGTATGTACAACGTGGAAGCCAAGCGACTGAACGATGAGGCTCGTAAAGAGGCAACACAGTCACGCGCTCTGGCGATTCGCTCCAACGAACTGGCTGACAGTGCATCCACTAAAGTTACCGAGGCTGCCCGTGGGGCAAACCAAGCCCAACAGCTTTCCAAATTCTTTGAGTAATCAAACA‑GGAGAAACCATTATGatcggttctgacatcgaagctaacgccct                                                                                                                                                                                                                                                                          >  1:85717‑M1/1‑272 (MQ=255)`  
                                        `aTTCTAATACGACTCACTAAAGGAGACACACCA‑TGTTCAAACTGATTAAGAAGTTAGGCCAACTGCTGGTTCGTATGTACAACGTGGAAGCCAAGCGACTGAACGATGAGGCTCGTAAAGAGGCCACACAGTCACGCGCTCTGGCGATTTGCTCCAACGAAATGGCTGACAGTGCATCCACTAAAGTTACCCAGGCTGCCCGTGTGGCAAACCAAGCTCAACAGCTTTCCAAATTCTTTGAGTAATCAAACA‑GGAGAAACCATCATGatagtttctgaaatcgaagctaacgccctcttag                                                                                                                                                                                                                                                                     >  1:16130‑M1/1‑267 (MQ=255)`  
                                        `aTTCTAATACGACTCACTAAAGGAGACACACCA‑TGTTCAAACTGATTAAGAAGTTAGGCCAACTGCTGGTTCGTATGTACAACGTGGAAGCCAAGCGACTGAACGATGAGGCTCGTAAAGAGGCCACACAGTCACGCGCTCTGGCGATTCGCTCCAACGAACTGGCTGACAGTGCATCCACTAAAGTTACCGAGGCTGCCCGTGTGGCAAACCAAGCTAAACAGCTTTCCAAATTCTTTGAGTCATCAAACA‑GGAGAAATCAATATGatcgtttctgacatcgaagctaacgccctcttag                                                                                                                                                                                                                                                                     >  1:121515‑M1/1‑267 (MQ=255)`  
                                         `ttCTAATACGACTCACTAAAGGAGACACACCA‑TGTTCAAACTGATTAAGAAGTTAGGCCAACTGCTGGTTCGTATGTACAACGTGGAAGCCAAGCGACTGAACGATGAGGCTCGTAAAGAGGCCACACAGTCACGCGCTCTGTCGCTTCGCTCCAACGAACTGGCTGACAGTGCATCCACTAAAGTTACCGAGGCTGCCCGTGTGGCAAACAAAGCTCAACAGCTTTCCAAATTCTTTGAGTAATCCAACA‑GGAGAAACCATTATGatcgtttctgacatcgaagctaacgccctcttcga                                                                                                                                                                                                                                                                    >  1:469765‑M1/1‑266 (MQ=255)`  
                                         `ttCTAATACGACTCACTAAAGGAGACACACCA‑TGTTCAAACTGATTAAGAAGTTAGGCCAACTGCTGGTTCGTATGTACAACGTGGAAGCCAAGCGACTGAACGATGAGGCTCGTAAAGAGGCCACACAGTCACGCGCTCTGGCGATTCGCTCCAACGAACTGGCTGACAGTGCATCCACTAAAGTTACCGAGGCTGCCCGTGTGGCAAACCAAGCTCAACAGCTTTCCAAATTCTTTGAGTAAT‑ACACACGGAGAAACCATTATGttcgttcatgacatcgaagcctgtcacttatacac                                                                                                                                                                                                                                                                    >  1:174629‑M1/1‑266 (MQ=255)`  
                                          `tataaaaagaatcaataaaggagACA‑ACCACAGTTCAAACTGATTAAAAAGTTAGGCCAACTGCTGGTTCGTAGGGACAACGTGGAAGCCAAGCGACTGAACGATGAGGCTCGTAAAGAGGCCACACAGTCACGCGCTCGGGCGATTCGCTCCAACGAAATGGCTGACAGTGAATCCACTAAAGTTACCGAGGCTGCCCGTGTGGCAAACCAAGCTCAACAGCTTTCCAAATGCTTTGAGTAATCAAACA‑GGAGAAACCATTATGatcgtttctgacatcgaagctaacgccctcttagag                                                                                                                                                                                                                                                                   <  1:57431‑M1/290‑37 (MQ=255)`  
                                          `tCTAATACGACTCACTAAAGGAGCCACACCA‑TGTTCAAACTAATTAAGAAGTTAGGCCAACTGCTGGTTCGTTTGTACAACGTAGAACCCAAGCGACTGACCGATGAGGCTCGTAAAGAGGCCACACAGTCACGCGCTCTGGCGATTCGCTCCAACGAACTGGCTGACAGTGCATCCACTAAAGTTACCGAGGCTGCCCGTGTGGCAAACCAAGCTCAACAGCTTTCCAAATTCTTTGAGTAATCAAACA‑GGAGAAATCAATATGatcgtttctgacatcgaagctaacgccctcttagag                                                                                                                                                                                                                                                                   <  1:363896‑M1/301‑37 (MQ=255)`  
                                          `agatgtgtataagagacagggagacacacca‑tgttcaaacTGATTAAGAAGTTAGGCCAACTGCTGGTTCGTATGTACAACGTGGAAGCCAAGCGACTGAACGATGAGGCTCGTAAAGAGGCCACACAGTCACGCGCTCTGGCGATTCGCTCCAACGAACTGGCTGACAGTGCATCCACTAAAGTTACCGAGGCTGCCCGTGTGGCAAACCAAGCTCAACAGCTTTCCAAATTCTTTGAGTAATCAAACA‑GGAGAAACCATTATGatcgtttctgacatcgaagctaacgccctcttagag                                                                                                                                                                                                                                                                   <  1:156501‑M1/282‑37 (MQ=255)`  
                                          `aataagagacagcactaaaggagacAAACCA‑TGTTCAAACTGATTAAGAAGTTAGGCCAACTGCTGGTTCGTATGTACAACGTGGAAGCCAAGCGACTGAACGATGAGGCTCGTAAAGAGGCCACACAGTCACGCGCTCTGGCGATTCGCTCCAACGAACTGGCTGACAGTGCATCCACTAAAGTTACCGAGGCTGCCCGTGTGGCAAACCAAGCTCAACAGCTTTCCAAATTCTTTGAGTAATCAAACA‑GGAGAAACCATTATGatcgtttctgacatcgaagctaacgccctcttagag                                                                                                                                                                                                                                                                   <  1:322800‑M1/289‑37 (MQ=255)`  
                                           `ataATACGACTCACTAAAGGAGAAACACCA‑TGTTCAAACTGATTAAGAAGTTAGGCCAACTGCTGGTGCGTATGTACAACGTGGAAGCCAAGCGACTGAACGATGAGGCTCGTAAAGAGGC‑‑CACAGTCACGCGCTCTGGCGATTCGCTCCAACGAACTGGCTGACAGGGCATCCACTAAAGTTACCGAGGCTGCCCGTGTGGCAAACCAAGCTCAACAGCTTTCCAAATTCTTTGAGTAATCAAACA‑GGAGAAACCATTATGatcgtttctgacatcgaagctaacgccctcttagagagc                                                                                                                                                                                                                                                                <  1:508937‑M1/300‑40 (MQ=255)`  
                                            `tAATACGACTCACTATAGGAGACACACCA‑TGTTCAAACTGATTAAGAAGTTAGGCCAACTGCTGGTTCGTATGTACAACGTGGAAGCCAAGCTACTGAACGATGAGGCTCGTAAAGAGGCCACACAGTCACGCGCTCTGGCGATTCGCTCCAACGAACTGGCTGACAGTGCATCCACTAAAGCTACCGAGGCTGCCCGTGTGGCAAACCAAGCTCAACAGCTTTCTAAAATCTTTGAGTAATCAAACA‑GGAGAAACCATTATGatcgtttctgatatagaagctaaagccctcttagagag                                                                                                                                                                                                                                                                 >  1:106304‑M1/1‑263 (MQ=255)`  
                                            `aaatACGACTCACTAAAGGAGACACACCA‑TGTTCAAACTGATTAAGAAGTTAGGCCAACTGCTGGTTCGTATGTACAACGTGGAAGCCAAGCGACTGAACGATGAGGCTCGTAAAGAGGCCACACAGTCACGCGCTCTGGCGATTCGCTCCAACGAACTGGCTGACAGTGCATCCACTAAAGTTACCGAGGCTGCCCGTGTGGCAAACCAAGCTCAACAGCTTTCCAAATTCTTTGAGTAATCAAACA‑GGAGAAATCAATATGatcgtttctgacatcgaagctaacgccctcttagagag                                                                                                                                                                                                                                                                 <  1:221909‑M1/300‑39 (MQ=255)`  
                                            `aaagagccagcactaaaggagACACACCA‑TGTTCAAACTGATTAAGAAGTTAGGCCAACTGCTGGTTCGTATGTAAAACGTGGAAGCCAAGCGACTGAACGATGAGGCTCGTAAAGAGGCCACACAGTCACGCGCTCTGGCGATTCGCACCAACGAACTGGCTGACAGTGCATCCACTAAAGTTACCGAGGCTGCCCGTGTGGCAAACCAAGCTCAACAGCTTTCCAAATTCTTTGAGTAATCAAACA‑GGAGAAACCATTATGatcgtttctgacatcgaagctaacgccctcttagagag                                                                                                                                                                                                                                                                 <  1:3526‑M1/291‑39 (MQ=255)`  
                                              `aTACGACTCACTAAAGGAGACACACCA‑TGTTCAAACTGATTAAGAAGTTAGGCCAACTGCTGGTTCGTATGTACAACGTGGAAGCCAAGCGACTGAACGATGAGGCTCGTAAAGAGGCCACACAGTCACGCGCTCTGGCGATTCGCTCCAACGAACTGGCTGACAGTGCATCCACTAAAGTTACCGACGCTGCCCGTGTGGCAAACCAAGCTCAACAGCTTTCCAAATTCTTTGAGTAATCAAACA‑GGAGAAACCATTAGGatcgtttctgacatcgaagcctgtctcttatacacatctc                                                                                                                                                                                                                                                               >  1:348256‑M1/1‑261 (MQ=255)`  
                                                  `gACTCACTAAAGGAGACACACCA‑TGTTCAAACTGATTAAGAAGTTAGGCCAACTGCTGGTTCGTATGTACAACGTGGAAGCCAAGCGACTGAACGATGAGGCTCGTAAAGAGGCCACACAGTCACGCGCTCTGGCGATTCGCTCCAACGAACTGGCTGACAGTGCATCCACTAAAGTTACCGAGGCTGCCCGTGTGGCAAACCAAGCTCAACAGCTTTCCAAATTCTTTGAGTAATCAAACA‑GGAGAAACCATTATGatcgtttctgacatcgaagctaacgccctcttagagagcgtcac                                                                                                                                                                                                                                                           <  1:238754‑M1/301‑45 (MQ=255)`  
                                                      `cACTAAAGGAGACACACCA‑TGTTCAAACTGATTAAGAAGTTAGGCCAACTGCTGGTTCGTATGTACAACGTGGAAGCCAAGCGACTGAACGATGAGGCTCGTAAAGAGGCCACACAGTCACGCGCTCTGGCGATTCGCTCCAACGAACTGGCTGACAGTGCATCCACTAAAGTTACCGAGGCTGCCCGTGTGGCAAACCAAGCTCAACAGCTTTCCAAATTCTTTGAGTAATCAAACA‑GGAGAAACCATTATGatcgtttctgacatcgaagctaacgccctcttagagctgtctcttata                                                                                                                                                                                                                                                       >  1:167779‑M1/1‑253 (MQ=255)`  
                                                      `cACTAAAGGAGACACACCA‑TGTTCAAACTGATTAAGAAGTTAGGCCAACTGCTGGTTCGTATGTACAACGTGGAAGCCAAGCGACTGAACGATGAGGCTCGTAAAGAGGCCACACAGTCACGCGCTCTGGCGATTCGCTCCAACGAACTGGCTGACAGTGCATCCACTAAAGTTACCGAGGCTGCCCGTGTGGCAAACCAAGCTCAACAACTTTCCAAATTCTTTGAGTAATCAAACA‑GGAGAAACCATTATGatcgtttctgacatcgaagctaacgccatcttagaaagcgtcactaag                                                                                                                                                                                                                                                       >  1:441121‑M1/1‑253 (MQ=255)`  
                                                       `aCTAAAGGAGACACACCA‑TGTTCAAACTGATTAAGAAGTTAGGCCAACTGCTGGTTCGTATGTACAACGTGGAAGCCAAGCGACTGAACGATGAGGCTCGTAAAGAGGCCACACAGTCACGCGCTCTGGCGATTCGCTCCAACGAACTGGCTGACAGTGCATCCACTAAAGTTACAGAGGCTGCCCGTGTGGCAAACCAAGCTCAACAGCTTTCCAAATTCTTTGAGTAATCAAACA‑GGAGAAACCATTATGatcgtttctgacatcgaagctaacgccctcttagagagcgtcactaaat                                                                                                                                                                                                                                                      >  1:143158‑M1/1‑252 (MQ=255)`  
                                                         `tAAAGGAGACACACCA‑TGTTCAAACTGATTAAGAAGTTAGGCCAACTGCTGGTTCGTATGTACAACGTGGAAGCCAAGCGACTGAACGATGAGGCTCGTAAAGAGGCCACACAGTCACGCGCTCTGGCGATTCGCTCCAACGAACTGGCTGACAGTGCATCCACTAAAGTTACCGAGGCTGCCCGTGTGGCAAACCAAGCTCAACAGCTTTCCAAATTCTTTGAGTAATCAAACA‑GGAGAAATCAATATGatcgtttctgacatcgaagctaacgccctcttagagagcgtcactaagttc                                                                                                                                                                                                                                                    <  1:396428‑M1/301‑52 (MQ=255)`  
                                                         `atgtgtaaaagatacagtgttcaaactgattaagaAGTGAGGCCAACTGCTGGTTCGTATGTACAACGTGGAAGCCAAGCGACTGAACGATGAGGCTCGTAAAGTGGCTACACAGTCACGCGCTCTGGCGATTCGCTCCAACGAACTGGCTGACAGTGCATCCACTAAAGTTACCGAGGCTGCCCGTGTGGCAAACCAAGCTAAACAGCTTTCCAAATTCTTTGAGTAATC‑AATA‑GGAGAAATCAATATGatcgtttctgacatcgaagctaacgccctcttagagagcgtcactaagttc                                                                                                                                                                                                                                                    <  1:480952‑M1/284‑52 (MQ=255)`  
                                                          `aaaGGAGACAAACCA‑TGTTCAAACTGATTAAGAAGTTAGGCAAACTGCTGGTTCGTATGTACAACGTGGAAGCAAAGCGACTGAACGATGAGGCTCGTAAAGAGGCCACACAGTCACGCGCTCTGGCGATTCGCTCCAACGAACTGGCTGACAGTGCATCCACTAAAGTTACCGAGGCTGCCCGTATGGCAAACCAAGCTCAACAGCTTTCCAAATTCTTTGAGTAATCAAACA‑GGAGAAACCATTATGatcgtttctgacatcgaagctaacgccctcttagagagcgtcactaagttcc                                                                                                                                                                                                                                                   <  1:116547‑M1/301‑53 (MQ=255)`  
                                                            `aGGAGACACACCA‑TGTTCAAACTGATTAAGAAGTTAGGCCAACTGCTGGTTCGTATGTACAACGTGGAAGCCAAGCGACTGAACGATGAGGCTCGTAAAGAGGCCACACAGTCACGCGCTCTGGCGATTCGCTCCGACGAACTGGCTGACAGTGCATCCACTAAAGTTACCGAGGCTGCCCGTGTGGCAAACCAAGCTCAACAGCTTTCCAAATTCTTTGAGTAATCAAACA‑GGAGAAACCATTATGatcgtttctgacatcgaagctaacgccctcttagagagcgtcactaagttccac                                                                                                                                                                                                                                                 <  1:311687‑M1/301‑55 (MQ=255)`  
                                                            `aGGAGACACACCA‑TGTTCAAACTGATTAAGAAGGTAGGCCAACTGCTGGTTCGTATGTACAACGTGGAAGCCAAGCGACTGAACGATGAGGCTCGTAAAGAGGCCACACAGTCACGCGCTCTGGCGTTTCGCTCCAACGAACTGGCTGACAGTGCATCCACTAAAGTTACCGAGGCTGCCCGTGTGGCAAACCAAGCTCAACAGCTTTCCAAATTCTTTGAGTAATCAAACA‑GGAGAAACCATTATGatcgtttctgacatcgaagctaacgacctcttagagagcgtcactaagttccac                                                                                                                                                                                                                                                 <  1:216308‑M1/301‑55 (MQ=255)`  
                                                                        `caggttcAAACTGATTAAGAAGTTAGGCCAACTGATGGTTCGTATGTACAACGTGGAAGCCAAGCGACTGAACGATGAGGCTCGTAAAGAGGCCACACAGTCACGCGCTCTGGCGATTCGCTCCAACGAACTGGATGACAGTGCATCCACTAAAGTTACCGAGGCTGCCCGTGTGGCAAACCAAGCTCAACAGCTTTCCACATTCTTTGAGTAATCAAACA‑GGAAGAATCAATATGatcgtttctgacatcgaatctaacgccctcttatagagcatcactaagttccacagcggggttat                                                                                                                                                                                                                                      >  1:294391‑M1/4‑236 (MQ=255)`  
                                                                         `ataagagacaggattaagaagttAGGCCAACTGCTGGTTCGTATGTACAACGTGGAAGCCAAGCGACTGAACGATGAGGCTCGTAAAGAGGCCACACAGTCACGCGCTCTGGCGATTCGCTCCAACGAACTGGCTGACAGTGCATCCACTAAAGTTACCGAGGCTGCCCGTGTGGCAAACCAAGCTCAACAGCTTTCCAAATTCTTTGAGTAATCAAACA‑GGAGAAATCAATATGatcgtttctgacatcgaagctaacgccctcttagagagcgtcactaagttccactgcggggttatc                                                                                                                                                                                                                                     <  1:453387‑M1/290‑67 (MQ=255)`  
                                                                            `ttttataagggacagaagttaggccaactgcTGGTTCGTATGTACAACGTGGAAGCCAAGCGACTTAACGATGAGGCTCGTAAAGAGGCCACACAGTCACGCGCTCTGGCGATTCGCTCCAACGAACTGGCTGACAGTGCATCCACTAAAGTTACCGAGGCTGCCCGTGTGGCAAACCAAGCTCAACAGCTTTCCAAATTCTTTGAGTAATCAAACA‑GGAGAAACCATTATGatcgtttctgacatcgaagctaacgccctcttagagagcgtcactaagttccactgcggggttatctac                                                                                                                                                                                                                                  <  1:32885‑M1/288‑70 (MQ=255)`  
                                                                               `aaaCTGATTAAGAGGTTAGGCCAACTGCTGGTTCGGATGTACAACGTGGAAGCCAAGCGACTGAACGATGAGGCTCGTAAAGAGGCCACACAGTCACGCGCTCTGGCGATTCGCTCCAACGAACTGGCTGACAGTGCATCCACTAAAGTTACCGAGGCTGCCCGTGTGGCAAACCAAGCTCAACAGCTTTCCAAATTCTTTGAGTAATCAAACA‑GGAGAAACCATTATGatcgtttctgacatcgaagctaacgccctcttagagagcgtcactaagttccactgcggggttatctacgac                                                                                                                                                                                                                               <  1:233935‑M1/301‑73 (MQ=255)`  
                                                                               `aaaCTGATTAAGAAGTTAGGCCAACTGCTGGTTCGTATGTACAACGTGGAAGCCAAGCGACTGAACGATTAGGCTCGTAAAGAGGCCACATAGTCACGCGCTCTGGCGATTCGCTCCAACGAACTGGCTGACAGTGCATCCACTAAAGTTACCGAGGCTGCCCGTGTGGCAAACCAAGCTCAACAGCTTTCCAAATTCTTTGAGTAATCAAACA‑GGAGAAACCATTATGatcgtttctgacatcgaagctaacgccctcttagagagcgtcactaagttccactgcggggttatctacgac                                                                                                                                                                                                                               <  1:72539‑M1/301‑73 (MQ=255)`  
                                                                                  `cTGATTAAGAAGTTAGGCCAACTGCTGGTTCGTATGTACAACGTGGAAGCCAAGCGACTGAACGATGAGGCTCGTAAAGAGGCCACACAGTCACGCGCTCTGGCGATTCGCTCCAACGAACTGGCTGACAGTGCATCCACTAATGTTACCGAAGCTGCCCGTGTGGCAAACCACGCTCAACAGCTTTCCAAATTCTTTGAGTAATCAAACA‑GGAGAAACAATTATGatcgtatcttacatcgaagctaacgccctcttagatagcgcccctaagttcccctgcggggttatctacgactac                                                                                                                                                                                                                            >  1:80947‑M1/1‑226 (MQ=255)`  
                                                                                    `gtataagtgataggggcaacggctggttcggatGTACAACGTGGAAGCCAAGCGACTGAACGATGGGGATCGTAAAGAGGCCACACAGTCACGCGCTCTGGCGATTCGCTCCAACGAACTGGCTGACAGTGCATCCACTAAAGTTACCGAGGCTGCCCGTGTGGCAAACCAAGCTCAACAGCTTTCCAAATTCTTTGAGTAATCAAACA‑GGAGAAACCATTATGatcgtttctgacatcgaagctaacgccctcttagagagcgtcactaagttccactgcggggttatctacgactactc                                                                                                                                                                                                                          <  1:27787‑M1/285‑78 (MQ=255)`  
                                                                                    `gATTAAGAAGTTAGGCCAACTGCTGGTTCGTATGTACAACGTGGAAGCCAAGCGACTGAACGATGAGGCTCGTAAAGAGGCCACACAGTCACGCGCTCTGGCGATTCGCTCCAACGAACTGGCTGACAGTGCATCCACTAAAGTTACCGAGGCTGCCCGTGTGGCAAACCAAGCTCAACAGCTTTCCAAATTCTTTGAGTAATCAA‑‑‑‑‑‑‑‑‑‑‑‑‑‑‑TATGatcgtttctgacatcgaagctaacgccctcttagagagcgtcactaagttccactgcggggttatctacgactactccaccgctgagtacg                                                                                                                                                                                                            >  1:263821‑M1/1‑210 (MQ=255)`  
                                                                                    `gATTAAGAAGTTAGGCCAACTGCTGGTTCGTATGTACAACGTGGAAGCCAAGCGACTGAACGATGAGGCTAGTAAAGAGGCCACACAGTCACGCGCTCTGGCGATTCGCTCCAACGAACTGGCTGACAGTGCATCCACTAAAGTTACCGAGGCTGCCCGTGTGGCAAACCAAGCTCAACAGCTTTCCAAATTCTTTGAGTAATCAAACA‑GGAGAAACCATTATGatcgtttctgacatccaagctaacgccctcttagcgagcgtcactaagttccactgcggggttatctacgactactc                                                                                                                                                                                                                          >  1:424482‑M1/1‑224 (MQ=255)`  
                                                                                        `aagaagGTAGGCCAACTGCTGGTTCGAATGAACAACGTAGAAGCCAAGCGACTGAACGATGAGGCTAGTAAAGAGGCCACACAGTCACGCGCTCTGGCGATTCGCTCCAACGAACTGGCTGACAGTGCATCCACTAAAGTTACCGAGGCTGCCCGTGTGGCAAACCAAGCTCAACAGCTTTCAAAATTCTTGGAGTAATCAAACA‑GGAGAAATAAATATGatcgtttctgacatcgaagctaacgccctcttagagagcgtcactaagttccactgcggggttatctacgactactccacc                                                                                                                                                                                                                      <  1:344273‑M1/301‑82 (MQ=255)`  
                                                                                          `gaagTTAGGCCAACTGCTGGTTCGTATGTACAACGTGGAAGCCAAGCGACTGAACGATGAGGCTCGTAAAGAGGCCACACAGTCATGCGCTCTGGCGATTCGCTCCAACGAACTGGCTGACAGTGCATCCACTAAAGTTACCGAGGCTGCCCGTGTGGCAAACCAAGCTCAACAGCTTTCCAAATTCTTTGAGTAATCAAACA‑GGAGAAACCATTATGatcgtttctgacatcgaagctaacgccctcttatagagcgtcactaagttccactgcggggttatctctgtctcttctccacc                                                                                                                                                                                                                    >  1:409155‑M1/1‑218 (MQ=255)`  
                                                                                          `gaagTTAGGCCAACTGCTGGTTCGTATGTACAACGTGGAAGCCAAGCGACTGAACGATGAGGCTCGTAAAGAGGCCACACAGTCACGCGCTCTGGCGATTCGCTCCAACGAACTGGCTGACAGTGCATCCACTAAAGTTACCGAGGCTGCCCGTGTGGCAAACCAAGCTCAACAGCTTTCCAAATTCTTTGAGTAATCAAACA‑GGAGAAACCATTATGatcgtttctgacatcgaagctaacgccctcttagagagcgtcactaagttccactgcggggttatctacgactactccaccgc                                                                                                                                                                                                                    <  1:208372‑M1/301‑84 (MQ=255)`  
                                                                                           `aagTTAGGCCAACTGCTGGTTCGTATGTACAACGTGGAAGCCAAGCGACTGAACGATGAGGCTCGTAAAGAGGCCACACAGTCACTCGCTCTGGCGATTCGCTCCAACGAACTGGCTGACAGTGCATCCACTAAAGTTACCGAGGCTGCCCGTGTGGCAAACCAAGCTCAACAGCTTTCCAAATTCTTTGAGTAATCAAACA‑GGAGAAACCATTATGatcgtttctgacatcgaagctaacgccctcttagagagcgtcactaagttccactgcggggttatctacgactactccaccgct                                                                                                                                                                                                                   >  1:304316‑M1/1‑217 (MQ=255)`  
                                                                                             `gTTAGGCCAACTGCTGGTTCGTATGTACAACGTGGAAGCCAAGCGACTGAACGATGAGGCTCGTAAAGAGGCCACACAGTCACGCGCTCTGGCGATTCGCTCCAACGAACTGGCTGACAGTGCATCCACTAAAGTTACCGAGGCTACCCGTGTGGCAAACCAAGCTCCACAGCTTTCCACATTCTTTGAGTAATCACACC‑AGAGAAAACATTATGatagtttctgacatcgaagctacagacctcttagagtgcgtcactaagttacaatgaggggttatctacgactactccaccgcttg                                                                                                                                                                                                                 >  1:260379‑M1/1‑215 (MQ=255)`  
                                                                                                    `gtataagagacaggtatgtacaacgtgGAAGCCAAGCGACTGAACGATGAGGCTCGTAAAGAGGCCACACAGTCACGCGCTCTGGCGATTCGCTCCAACGAACTGGCTGACAGTGCATCCACTAAAGTTACCGAGGCTGCCCGTGTGGCAAACCAAGCTCAACAGCTTTCCAAATTCTTTGAGTAATCAAACA‑GGAGAAACCATTATGatcgtttctgacatcgaagctaacgccctcttagagagcgtcactaagttccactgcggggttatctacgactactccaccgctgagtacgta                                                                                                                                                                                                          <  1:146934‑M1/288‑94 (MQ=255)`  
                                                                                                                `cGTATGTACAACGTGGAAGCCAAGCGACTGAACGATGATGCTCGTAAAGAGGACACACAGTCACGCGCTCTGGCGATTCGCTCCAACGAACTGGCTGACAGTGCCTCCACTAAAGTTACCGAGGCTTCCCGTGTTGCAAACCAAGCTCAACAGCTTTCCAAATTCTTTGAGTAATAAAACA‑GGAGAAACCATTATGatcgtttctgacatcgaagataacgccctcttagagatcgtcactaagttccacttcggggttatctacgaatactccaccgctgagtacctgtctcttatacaa                                                                                                                                                                                              >  1:339340‑M1/1‑196 (MQ=255)`  
                                                                                                                `cGTATGTACAACGTGGAAGCCAAGCGACTGAACGATGAGGCTCGTAAAGAGGCCACACAGTCACGCGCTCTGGCGATTCGCTCCAACGAACTGGCTGACAGTGCATCCACTAAAGTTACCGAGGCTGCCCGTGTGGCAAACCAAGCTCAACAGCTTTCCAACTTCTTTGAGTAATCAAACA‑GGAGAAACCATTATGatcgtttctgacatcgaagctaacgccctcttagagggcgtcactaagttccactgcggggttatctacgactactccaccgctgcgtacgtaagctaccgtccc                                                                                                                                                                                              >  1:405785‑M1/1‑196 (MQ=255)`  
                                                                                                                  `tATGTACAACGTGGAAGCCAAGCGACTGAACGATGAGGCTCGTAAAGAGGCCACACAGTCACGCGCTCTGGCGATTCGCTCCAACGAACTGGCTGACAGTGCATCCACTAAAGTTACCGAGGCTGCCCGTGTGGCAAACCAAGCTCAACAGCTTTCCAAATTCTTTGAGTAATCAAACA‑GGAGAAACCATTATGatcgtttctgacatcgaagctaacgccctcttagagagcgtcactaagttccactgcggggttatctacgactactacaccgctgagtacgtaagctacctgtctct                                                                                                                                                                                            >  1:24441‑M1/1‑194 (MQ=255)`  
                                                                                                                                   `ccAAGCGACTGAACGATGAGGCTCGTAAAGAGGCCACACAGTCACGCGCTCTGGCGATTCGCTCCAACGAACTGGCTGACAGTGCATCCACTAAAGTTACCGAGGCTGCCCGTGTGGCAAACCAAGCTCAACAGCTTTCCAAATTCTTTGAGTAATCAAACA‑GGAGAAACCATTATGatcgtttctgacatcgaagctaacgccctcttagagagcgtcactaagttccactgcggggttatctacgactactccaccgctgagtacgtaagctaccgtccgagtgacttcggtgcgtatc                                                                                                                                                                           >  1:139394‑M1/1‑177 (MQ=255)`  
                                                                                                                                          `aCTGAACGATGAGGCTCGTAAAGAGGCCACACAGTCACGCGCTCTGGCGATTCGCTCCAACGAACTGGCTGACAGTGCATCCACTAAAGTTACCGAGGCTGCCCGTGTGGCAAACCAAGCTCAACAGCTTTCCAAATTCTTTGAGTAATCAAACA‑GGAGAAACCATTATGatcgtttctgacatagaagctaacgccctcttagagagcgtcactaagttccactgcggggttatctacgactactcaaccgctgagtacgtaagctaccgtccgagtgacttcggtgcgtatctggaagc                                                                                                                                                                    >  1:272545‑M1/1‑170 (MQ=255)`  
                                                                                                                                             `gAACGATGAGGCTCGTAAAGAGGCCACACAGTCACGCGCTCTGGCGATTCGCTCCAACGAACTGGCTGACAGTGCATCCACTAAAGTTACCGAGGCTGCCCGTGTGGCAAACCAAGCTCAACAGCTTTCCAAATTCTTTGAGTAATCAAACA‑GGAGAAACCATTATGatcgtttctgacatcgaagctaacgccctcttagagagagtcgctaagttccactgcggggttctctacgactactccaccgctgagtacgtaagctacagtccgagttacttcggtgcgtatctggatgcgct                                                                                                                                                                 >  1:373740‑M1/1‑167 (MQ=255)`  
                                                                                                                                                        `cTCGTAAAGAGGCCACACAGTCACGCGCTCTGGCGATTCGCTCCAACGAACTGGCTGACAGTGCATCCACTAAAGTTACCGAGGCTGCCCGTGTGGCAAACCAAGCTCAACAGCTTTCCAAATTCTTTGAGTAATCAAACA‑GGAGAAATCAATATGatcgtttctgacatcgaagctaacgccctcttagagagcgtcactaagttccactgcggggttatctacgactactccaccgctgagtacgtaagctaccgtccgagtgacttcggtgcgtatctggatgcgctggatgccgaag                                                                                                                                                      >  1:269318‑M1/1‑156 (MQ=255)`  
                                                                                                                                                          `cGTAAAGATGCCACACAGTCACGCGCTCTGGCGATTCGCTCCAACGAACTGGCTGACAGTGCATCCACTAAAGTTACCGAGGCTGCCCGTGTGGCCAACCAAGCTCAACAGCTTTCCAAATTCTTTGAGTAATCAAACA‑GGAGAAACCATTATGatcgtttctgacatcgaagctaacgccctcttagagagcgtcactaagttccactgcggggttatctacgactactccaccgctgagtacgtaagctaccgtccgagtgacttcggtgcgtatctggatgcgctggaagccgcggtt                                                                                                                                                    >  1:487146‑M1/1‑154 (MQ=255)`  
                                                                                                                                                              `aaGGGGACACACAGTCACGCGCTCTGGCGATTCGCGTCAACGAACTGGCGGACAGTGCATCCACTAAAGTTACCGAGGCTGCCCGTGTGGCAAACCAAGCTCAACAGCTTTCCAAATTCTTTGAGTAATCAAACA‑GGAGAAACCATTATGatcgtttctgacatcgaagctaacgccctcttagagagcgtcactaagttccactgcggggttatctacgactactccaccgctgagtacgtaagcgaccgtccgagtgacttcggtgcgtatctggatgcgctggaagccgaggttgcac                                                                                                                                                <  1:46215‑M1/301‑152 (MQ=255)`  
                                                                                                                                                                         `caGTCACGCGCTCTGGCGATTCGCTCCAACGAACTGGCTGACAGTGCATCCACTAAAGTTACCGAGGCTGCCCGTGTGGCAAACCAAGCTCAACAGCTTTCCAAATTCTTTGAGTAATCAAACA‑GGAGAAATCAATATGatcgtttctgacatcgaagctaacgccctcttagagagcgtcactaagttccactgcggggttatctacgactactccaccgctgagtacgtaagctaccgtccgagtgacttcggtgcgtatctggatgcgctggaagccgaggttgcacgaggcggtctt                                                                                                                                     >  1:199549‑M1/1‑139 (MQ=255)`  
                                                                                                                                                                         `caGTCACGCGCTCTGGCGATTCGCTCCAACGAACTGGCTGACAGTGCATCAACTAAAGTTACCGAGGCTGCCCGTGTGGCAAACCAAGCTCAACAGCTTTCCAAATTCTTTGAGTAATCAAACA‑GGAGAAACAATTATGatcgtttctgacatcgaagctaacgccctcttagagagcgtcactaagtacaactgcggggttatctacgactactccaccgctgaatacgtaagatacagtccgagtgacttcggtgcgaatctggaggcgcgggaagacgaggttgaactaggaggtctt                                                                                                                                     >  1:256198‑M1/1‑139 (MQ=255)`  
                                                                                                                                                                           `gTCGCGCGCTCGGGCTATGCGCTCCAACGAACTGTCTGACAGTGCATCCACTAAAGTCACCGAGGCTGCCCGTGTGGCAAACCAATCTCAACAGCTTTCCAAATTCTTTGAGTAATCAAACA‑GGAGAAATCAAGATGttcgtttctgacatcgaagctaacgccctcttagagagcgtcactaagttccactgcggggttatctacgactactccaccgctgagtacgtaagctaccgtccgagtgacttcggtgcgtatctggatgcgctggaagccgaggttgcacgaggcggtcttat                                                                                                                                   <  1:204622‑M1/301‑165 (MQ=255)`  
                                                                                                                                                                           `gTCACGCGCTCTGGCGATTCGCTCCAACGAACTGGCTGACAGTGCATCCACTAAAGTTACCGAGGCTGCCCGTGTGGCAAACCAAGCTCAACAGCTTTCCAAATTCTTTGAGTAATCAAACA‑GGAGAAACCATTATGatcgtttctgacatcgaagctaacgccctcttagagagcgtcactaagttccactgcggggttatctacgactactccaccgctgagtacgtaagctaccgtccgagtgacttcggtgcgtatctggatgcgctggaagccgaggttgcacgaggcggtcttat                                                                                                                                   >  1:152016‑M1/1‑137 (MQ=255)`  
                                                                                                                                                                             `cACGCGCTCTGGCTCTTCGCTCCACCGAACTGGCTGACAGTGCATCCACTATCGTTACCGAGGCTGCTCGTGTGGCAAACCAAGCTCAACAGCTTTCCAAATTCTTTTAGTAATCCAACA‑GGAGAAACCATTATGatcgtttctgacatcgaagctaactccctcttagagagcgtcactatgttccactgcggggttatctacgactactccaccgcttagtacgtaagctaccgtccgagtgacttcggtgcgtatctggatgcgctggaagccgaggttgcacgatgcggtcttattg                                                                                                                                 <  1:504134‑M1/301‑167 (MQ=255)`  
                                                                                                                                                                             `cACGCGCTCTGGCGATTCGCTCCAACGAACTGGCGGACAGTGCATCCACTAAAGTTACCGAGGCTGCCCGTGTTGCAAACCAAGCTCAACAGCTTTCCAAATTCTTTGAGTAATCAAACA‑GGAGAAACCATTATGatcgtttctgacatcgaagctaacgccctcttagagagcgtcactaagttccactgcggggttatctacgactacgccaccgctgagtacgtaagctaccgtccgagtgaattcggtgcgtatctggatgcgctggaagccgaggttgcacgaggcggtattattg                                                                                                                                 <  1:253853‑M1/301‑167 (MQ=255)`  
                                                                                                                                                                               `cgcgCTCTGGCGATTCGCTCCAACGAACTGGCTGACAGTGCATCCACTAAAGTTACCGAGGCTGCCCGTGTGGCAAACCAAGCTCAACAGCTTTCCAAATTCTTTGAGTAATCAAACA‑GGAGAAACCATTATGatcgtttctgacatcgaagctaacgccctcttagagagcgtcactaagttccactgcggggttatctacgactactccaccgctgagtacgtaagctaccgtccgagtgacttcggtgcgtatctggatgcgctggaagccgaggttgcacgagtcggtcttattgtg                                                                                                                               <  1:357163‑M1/301‑169 (MQ=255)`  
                                                                                                                                                                                   `ctctGGCGATTCGCTCCAACGAACTGGCTGACAGTGCATCCACTAAAGTTACCGAGGCTGCCCGTGTGGCAAACCAAGCTCAACAGCTTTCCAAATTCTTTGAGTAATCAAACA‑GGAGAAACCATTATGatcgtttctgacatcgaagctaacgccctcttagagagcgtcactaagttccactgcgtggttatctacgactactccaccgctgagtacgtaagctaccgtccgagtgacttcggtgcgtatctggatgcgctggaagccgaggttgcacgaggcggtcttattgtgttcc                                                                                                                           >  1:91467‑M1/1‑129 (MQ=255)`  
                                                                                                                                                                                   `ctctGGCGATTCGCTCCAACGAACTGGCTGACAGTGCATCCACTAAAGTTACCGAGGCTGCCCGTGTGGCAAACCAAGCTCAACAGCTTTCCAAATTCTTTGAGTAATCAAACA‑GGAGAAACCATTATGatcgtttctgacatcgaagctaacgacctcttagagagcgtcactaagttccactgcggggttatctacgactactccaccgctgagtacgtaagctaccgtccgagtgacttcggtgcgtatctggatgcgctggaagccgaggttgcacgaggcggtcttattgtgttcc                                                                                                                           >  1:428437‑M1/1‑129 (MQ=255)`  
                                                                                                                                                                                       `ggCGATTCGCTCAAACGAACTGGCTGACAATTGATCCACTAAATTTACCGAGGCTGCCCGTGTGGCAAACCAAGCTCAACAGCTTTCCAAATTCTTTGAGTAATCAAACA‑GGAGAAACCATTATGatcttttctgacatcgaagctaacgccctcttagagagcgtcactaagttccactgcggggttatctacgactactccaccgctgagtacgtaagctaccgtccgagtgacttcggtgcgtatctggatgcgctggaagccgaggttgcacgaggcggtcttattgtgttccacaa                                                                                                                       <  1:62582‑M1/301‑177 (MQ=255)`  
                                                                                                                                                                                                       `aaacTGGCTGACAGTGCATCCACTAAAGTTACCGAGGCTGCCCGTGTGGCAAACCAAGCTCAACAGCTTTCCAAATACTTGGAGTAATCAAACA‑GGAGAAAGCAATATGctcgtttttgacatcgaagctaacgccctcttagatagcgtcactaagtcccactgcggggttatctacgactactccaccgctgagtacgtaatctaccgtccgagtgacttcgatgcgtatctggatgcgctggaagccgaggttgcacgaggcggtcttattgtgttccacaacggtcacaagtatgac                                                                                                       <  1:95322‑M1/300‑193 (MQ=255)`  
                                                                                                                                                                                                        `acagggaagacagggcaTCCAGTAAAGATACCGAGGATGACCGTGTGGCAAACAAAGCTCAACAGCATTCCAAAATCATAGAGTAATCAAACA‑AGAGAAAACAGTATGatcgtttctgacatcgaagctaacgccctcttagagagcgtcactaagttccactgcggggttatctacgactacgccaccgctgagtacgtaagctaccgtccgagtgacttcggtgcgtatctggatgcactggaagccgaggttgcacgaggcggtcttattgtgttccacaacggtcacaagtatgaca                                                                                                      <  1:457494‑M1/293‑194 (MQ=255)`  
                                                                                                                                                                                                        `aaCTGGCTGACAGTGCATCCACTAAAGTTACCGAGGCTGCCCGTGTGGCAAACCAAGCTCAACAGCTGTCCAAATTCTTTGAGTAATCAAACA‑GGAGAAACCATTATGatcgtttctgacatctaagctaacgccctcttagagagcgtcactaagttccactgcggggttatctacgactactccaccgctgagtacgtaagctaccgtccgagtgacttcggtgcgtatctggatgcgctggaagccgaggttgcacgaggcggtcttattgtgttccacaacggtcacaagtatgacg                                                                                                      <  1:436813‑M1/301‑194 (MQ=255)`  
                                                                                                                                                                                                        `aaCTGGCTGACAGTGCATCCACTAAAGGTACCGAGGCTGCCCGTGTGGCAAACCAAGCTCAACAGCTTTCCAAATTCTTTGAGTAATCAAACA‑GGAGAAACCATTATGatcgtttctgacatcgaatctaacgccctcttagagagcgtcactaagttccactgcggggttatctacgactactccaccgctgagtacgtaagctaccgtccgagtgacttcggtgcgtatctggatgcgctggaagccgaggttgcacgaggcggtcttattgtgttccacaacggtcacaagtatgacg                                                                                                      <  1:387544‑M1/301‑194 (MQ=255)`  
                                                                                                                                                                                                         `aCTGGCTGACAGTGCAACCACTAAAGTTACCGAGGCTGCCCGTGTGGCAAACCAAGCTCAACAGCTTTCCAAATTCTTTGAGTAATCAAACA‑GGAGAAACCATTATGatcgtttctgacatcgaagctaacgccctcttagagagcgtcactaagttccactgcggggttatctacgactactccaccgctgagtacgtaagctaccgtccgagttacttcggtgcgtatctggatgcgcaggaagccgaggttgcacgaggcggtcttattgtgttccacaacggtcacaagtatgacgt                                                                                                     <  1:465140‑M1/301‑195 (MQ=255)`  
                                                                                                                                                                                                              `cTGACAGTGCATCCACTAAAGTTACCGAGGCTGCCCGTGTGGCAAACCAAGCTCAACAGCTTTCCAAATTCTTTGAGTAATCAAACA‑GGAGAAACCATTATGatcgtttctgacatcgaagctaacgccctcttagagagcgtcactaagttccactgcggggttatctacgactactccaccgctgagtacgtaagctaccgtccgagtgacttcggtgcgtatctggatgcgctggaagccgaggttgcacgaggcggtctcattgtgttccacaacggtcacaagtatgacgttcctg                                                                                                <  1:377421‑M1/301‑200 (MQ=255)`  
                                                                                                                                                                                                                 `caggtgcATCCACTAAAGTTACCGAGGCTGCCCGTGTGGCAAACCAAGCTCAACAGCTTTCCAAATTCTTTGAGTAATCAAACA‑GGAGAAACCATTATGatcgtttctgacatcgaagttaacgccctcttagagagcgtcactaagttccactgctgggttatctacgactactccaccgctgagtacgtaagctaccgtccgagtgacttcggtgcgtatctggatgcgctggaagccgaggttgcacgagtcggtcttattgtgttccacaacggtcacaagtatgacgttcctgcat                                                                                             <  1:190587‑M1/298‑203 (MQ=255)`  
                                                                                                                                                                                                                  `cAGTGCATCCACTAAAGTTACCGAGGCTGCCCGTGTGACAAACCAAGCTCAACAGCTTTCCAAATTCTTTGAGTAATCAAACA‑GGAGAAACCATTATGatcgtttctgacatcgcagctaacgccctcttagagagcggcactaagttccactgcggggttatctacgactactccaccgctgagtacgtaagctaccgtccgagtgacttcggtgcgtatctggatgcgctggaagccgcggttgcacgaggccgtcttattgttttccccaacggtcgcaagtatgacgttccttcatt                                                                                            >  1:101400‑M1/1‑98 (MQ=255)`  
                                                                                                                                                                                                                   `aGTGCATCCACTAAAGTTACCGAGGCTTCCCGTGTGGCAAACCAAGCTCAACAGCTTTCCAAATTCTTTGAGTAATCAAACA‑GGAGAAACCATTATGatcgtttctgacatcgaagctaacgccctcttagagagcgtcactaagttccactgcggggttatctacgactactccaccgctgagtacgtaagctaccgtccgagtgacttcggtgcgtatctggatgcgctggaagccgaggttgcacgaggcggtcttattgtgttccacaacggtcacaagtatgacgttcctgcattg                                                                                           <  1:474350‑M1/301‑205 (MQ=255)`  
                                                                                                                                                                                                                      `gCATCCACTAAAGTTACCGAGGCTGCCCGTGTGGCAAACCAAGCTCAACAGCTTTCCAAATTCTTTGAGTAATCAAACA‑GGAGAAATCAATATGatcgtttctgacatcgaagctaacgcccgcttagagagcgtcactaagttccactgcggggttatctacgactactccaccgctgagtacgtaagctaccgtacgagtgacttcggtgcgtatctggatgcgctggaagccgaggttgcatgaggcggtcttattgtgttccacaacggtcacaagtatgacgttcctgcattgacc                                                                                        <  1:130625‑M1/301‑208 (MQ=255)`  
                                                                                                                                                                                                                      `gCATCCACTAAAGTTACCGAGGCTGCCCGTGTGGCAAACCAAGCTCAACAGCTTTCCAAATTCTTTGAGTAATCAAACA‑GGAGAAACCATTATGatcgtttctgacatcgaagctaacgccctcttagagagcgtcactaagttccactgcggggttatctacgactactccaccgctgagtacgtaagctaccgtccgagtgacttcggtgcgtatctggatgcgctggaagccgaggttgcacgaggcggtcttattgtgttccacaacggtcacaagtatgacgttcctgcattgacc                                                                                        >  1:113490‑M1/1‑94 (MQ=255)`  
                                                                                                                                                                                                                          `ccACTAAAGTTACCGAGGCTGCCCGTGTGGCAAACCAAGCTCAACAGCTTTCCAAATTCTTTGAGTAATCAAACA‑GTAGAAACCATTATGatcgtttctgacatcgaagctaacgccctcttagagagcgtcactaagttccactgcggggttatctacgactactccaccgctgagtacgtaagctaccgtccgagtgacttcggtgcgtatctggatgcgctggaagccgaggctgcacgaggcggtcttattgtgttccacaacggtcacaagtatgacgttcctgcattgaccaaac                                                                                    >  1:6704‑M1/1‑90 (MQ=255)`  
                                                                                                                                                                                                                           `cACTAAAGTTACCGAGGCTGCCCGTGTGGCAAACCAAGCTCAACAGCTTTCCAAATTCTTTGAGTAATCAAACA‑GGAGAAACCATTATGatcgtttctgacatcgaagctaatgccctcttagagagcgtcactaagttccactgcggggttatctacgactactccaccgctgagtacgtaagataccgtccgagtgacttcggtgcgtatctggatgcgatggaagccgaggttgcacgcggcggtcttattgtgttccacaacgggcacaagtatgacgtgccggcatctgtcgctaa                                                                                   >  1:433254‑M1/1‑89 (MQ=255)`  
                                                                                                                                                                                                                                   `ttACCGAGGCTGGCCGTGTGGCAAACCAAGCTCAACAGCTTTCCGAATTCTTTGAGTAATCAAACA‑GGATAAACCATTATGatcgtttctgacatcgaagctaacgccctcttagagagcgtaactaaggtccactgcggggttatctacgactactccaccgctgagtacgtaagctaccgtccgagtgacttcggtgcgtatctggatgcgctggaagccgaggttgaacgaggcggtcttattgtgttccacaacggtcacaagtatgacgttcctgcattgaccaaactggcaaagt                                                                           <  1:248282‑M1/301‑221 (MQ=255)`  
                                                                                                                                                                                                                                    `ttccgaGGCTGCCCGTGTGGCAAACCAAGCTCAACAGCTTTCCAAATTCTTTGAGTAATCAAACA‑GGAGAAATCAATATGatcgtttctgacatcgaagctaacgccctcttagagagcgtaactaagttccactgcggggttatctacgactactccaccgctgagtacgtaagctaccgtccgagtgacttcggtgcgtatctggatgcgctggaaaccgaggttgcacgaggcggtcttattgtgttccacaacggtcacaagtatgccgttcctgcattgaccctgtctcttataca                                                                          >  1:194538‑M1/3‑80 (MQ=255)`  
                                                                                                                                                                                                                                              `gCCCGTGTGGCAAACCAAGCTCAACAGCTTTCCAAATTCTTTGAGTAATCAAACA‑GGAGAAATCAATATGatcgtttctgacatcgaagctaacgccctcttagagagcgtcactaagttccactgcggggttatctccgactactccaccgctgagtacgtaagctaccgtccgagtgacttcggtgcgtatctggattcgctggaagccgaggttgcacgaggcggtcttattgttttccacaacggtcacaagtataacgttcctgcattgaccaaacctgtctcttctacacatctc                                                                >  1:231245‑M1/1‑70 (MQ=255)`  
                                                                                                                                                                                                                                                     `gtgggctaggatatgtgtataagagacagttctttgagtaatcaaaca‑ggagaaactattATGatcgtttctgacatcgcagctagcgccctcttagagagcgtcactaagttccactgcggggttatctacgacttctccaccgctgattacgtaagctaccgtccgagtgacttcggtgcgtatctggatgcgctggaagccgaggttgcacgaggcggtcttattgtgttccacaacggtcacaagtatgacgttcctgcattgaccaaactggcaaagttgcaattgaaccgagagt                                                         <  1:382934‑M1/272‑239 (MQ=255)`  
                                                                                                                                                                                                                                                      `ggCAAACCAAGCTCAACAGCTTTCCAAATTCTTTGAGTAATCAAACA‑GGAGAAACCATTATGatcgtttctgacatcgaagctaacgccctcttagagagcgtcactaagttccactgcggggttatctacgactactccaccgctgagtacgtaagctaccgtccgagtgacttcggtgcgtatctggatgcactggaagccgaggttgcacgaggcggtcttattgtgttcaacaacggtcacaactatgacgttcctgcattgaccaaactggccacgttgccattcaaccgagagtt                                                        >  1:368946‑M1/1‑62 (MQ=255)`  
                                                                                                                                                                                                                                                          `aaCCAAGCTCAACAGCTTTCCAAATTCTTTGAGTAATCAAACA‑GGAGAAACCATTATGatcgtttctgacatcgaagctaacgccctcttagagagcgtcactaagttccactgcggggttatctacgactactccaccgctgagtacgtaagctaccgtccgagtgacttcggtgcgtatctggatgcgctggaagccgaggttgcacgaggcggtcttattgtgttccacaacggtcacaagtatgacgttcctgcattgaccaaactggcaaagttgcaattgaaccgagagttccac                                                    <  1:419663‑M1/301‑244 (MQ=255)`  
                                                                                                                                                                                                                                                           `aCCAAGCTCAACAGCTTTCCAAATTCTTTGAGTAATCAAACA‑GGAGAAACCATTATGatcgtttctgacatcgaagctaacgccctcttagagagcgtcactaagttccactgcggggttatctacgactactccaccgctgagtacgtaagctaccgtccgagtgacttcggtgcgtatctggatgcgctggaagccgaggttgcacgcggcggtcttattgtgttacacaacggtcacaagtatgacgttcctgcattgaccaaactggcaaagttgccattgcaccgagagttccacc                                                   >  1:211933‑M1/1‑57 (MQ=255)`  
                                                                                                                                                                                                                                                            `ccAAGCTCAACAGCTTTCCAAATTCTTTGAGTAATCAAACA‑GGAGAAACCATTATGctcgtttctgacatcgaagctaacgccctcttagagagcgtcactaagttccactgcggggttatctacgactactccaccgctgagtacgtaagctaccgtccgagtgacttcggtgcgtatctggatgcgctggaagccgaggttgcacgaggcggtcttattgtgttccacaacggtcacaagtatgacgttcctgcattgaccaaactggcaaagttgcaattgaacagagagttccacct                                                  <  1:179487‑M1/301‑246 (MQ=255)`  
                                                                                                                                                                                                                                                              `aaGCTCACTATCTTTCCAAATTCTTTGAGTAATCACACA‑GTAGAAATCAATATGatcgtttctgacatcgaagctaacgccctcttagagagcgtcactaagttccactgcgtggttatctacgactactccaccgctgagtacgtaagctaccgtccgagtgacttcggtgcgtatctggattcgctggaagccgaggttgcacgaggcggtcttattgtgttccacaacggtcacaagtatgacgttcctgcattgaccaaactggcaaagttgcaattgaaccgagagttccaccttc                                                <  1:126490‑M1/301‑248 (MQ=255)`  
                                                                                                                                                                                                                                                                `gCTCAACAGCTTTCCAAATTCTTTGAGTAATCAAACA‑GGAGAAACCATTATGatcgtttctgacatcgaagctaacgccctcttagagagcgtcactaagttccacttcggggttatctacgactactccaccgctgagtacgtaagctaccgtccgagtgacatcggtgcgtatctggttgctctggaagccgaggttgcacgaggcggtcttattgtgttccacaacggtcccaagtatgacgttcctgcattgaccaaactggcaaagttgcaattgaaccgagagttccaccttccc                                              >  1:400740‑M1/1‑52 (MQ=255)`  
                                                                                                                                                                                                                                                                 `atttgtataagttactgttctttgagtaatcaaaca‑GGAGAAACCATTATGatcgtttctgacatcgaagctaacgccctcttagagatcgtcactaagttccactgctgggttatctacgactactccaccgctgagtacgtaagctaccgtccgagtgacttcggtgcgtatctggatgcgctggaagccgaggttgcacgaggcggtcttattgtgttccacaacggtcacaagtatgacgttcctgcattgaccaaactggcaaagttgcaattgaaccgagagttccaccttcctc                                             <  1:109918‑M1/284‑251 (MQ=255)`  
                                                                                                                                                                                                                                                                  `tCAACAGCTTTCCAAATTCTTTGAGTAATCAAACA‑GGAGAAACAATTATGatcgttgctgacatcgaagctaactccctcttagagagcgtcactaagttccactgcggggttatctacgactactccaccgctgagtacgtaagctaccgtccgattgacttcggtgcgtatctggatgcgctggaagccgaggttgcacgaggcggtcttattgtgttccacaacggtcacaagtatgacgttcctgcattgaccaaactggcaaagttgcaattgaaccgagagttccaccttcctcg                                            <  1:225266‑M1/301‑252 (MQ=255)`  
                                                                                                                                                                                                                                                                           `ttCCAAATTCTTTGAGTAATCAAACA‑GGAGAAACCATTATGatcgtttctgacatcgaagctaacgccctcttagagagcgtcactaagttccactgcggggttatctacgactactccaccgctgagtacgtaagctaccgtccgagtgacttcggtgcgtatctggatgcgctggaagccgaggttgcacgaggcggtcttattgtgttccacaacggtcacaagtatgacgatcctgcattgaccaaactggcaaagttgcaattgaaccgagagttcaaccttcctcgtgctgtctc                                   >  1:493616‑M1/1‑41 (MQ=255)`  
                                                                                                                                                                                                                                                                                `aaTTCTTTGAGTAATCAAACA‑GGAGAAACCATTATGatcgtttctgacatcgaagctaacgccctcttagagagcgtcactaagttccactgcggggttatctacgactactccaccgctgagtacgtaagctaccgtccgagtgacttcggtgcgtatctggatgagctggaagccgaggttgcacgaggcggtcttattgtgttccacaacggtcacaagtatgacgttcctgcattgaccaaactggcaaagttgcaatgtaaccgagagttacaccttcctcgtgagaactgtattt                              >  1:333276‑M1/1‑36 (MQ=255)`  
                                                                                                                                                                                                                                                                                 `aTTCTTTGAGTAATCAAACA‑GGAGAAACCATTATGatcgtttctgacatcgaagctaacgccctcttagagagcgtcactaagttccactgcggggttatctacgactactccaccgctgagtacgtaagctaccgtccgagtgacttcggtgcgtatctggatgcgctggaaaccgaggttgcacgaggcggtcttattgtgttccacaacggtcacaagtatgacgttcctgcattgaccaaactggcaaagttgcaattgaaccgagagttccaccttcctcgtgagaactgtattga                             <  1:316042‑M1/301‑267 (MQ=255)`  
                                                                                                                                                                                                                                                                                   `aagacaggtaatcaaACA‑GGAGAAACCATTATGatcgtttctgacatcgaagctaacgccctcttagagagcgtcactaagttccactgcggggttatctacgactactccaccgctgagtacgtaagctaccgtccgagtgacttcggtgcgtatctggatgcgctggaagccgaggttgcacgaggcggtcttattgtgttccacaacggtcacaagtatgacgttcctgcattgaccaaactggcaaagttgcaattgaaccgagagttccaccttcctcgtgagaactgtattgaca                           <  1:414140‑M1/294‑269 (MQ=255)`  
                                                                                                                                                                                                                                                                                        `gAGTAATCAAACA‑GGAGAAACCATTATGatcgtttctgacatcgaagctaacgccctcttagagagcgtcactatgttccacttcggggttatctacgactactccaccgctgagtacgtaagctaccgtccgagtgacttcggtgcgtatctggatgcgctggaagccgaggttgcacgaggcggtcttattgtgttccacaacggtcacaagtatgacgttcctgcattgaccaaactggcaaagttgcaattgaaccgagagttccaccttcctcgtgagaactgtattgataccctt                      <  1:299544‑M1/301‑274 (MQ=255)`  
                                                                                                                                                                                                                                                                                            `aaTCAAACA‑GGAGAAACCATTATGatcgtttctgacatcgaagctaacgccctcttagagagcgtcactaagttcaactgcggggttatctacgactactccacagctgagtacgtaagctaccgtccgagtgacttcggtgcgtatctggatgcgctggaagccgagtttgcacgaggcggtcttattgtgttccacaacggtcacaagtctgacgttcctgcattgaccaaactggcaaagttgcaattgaaccgagagttccacattcctcgtgagacctgtatagaaacccttgtgt                  >  1:52909‑M1/1‑24 (MQ=255)`  
                                                                                                                                                                                                                                                                                                `aaaCA‑GGAGGAACCATTAGGatcgtttctgacatcgaagctaacgcagtcttagagagcgtcactaagttccactgcggggttatctacgactactcaaccgctgagtacgtaagctacagtccgagtgacttcggtgcgtatctgtatgcgatggaagccgaggttgcacgaggcggtcttattgtgttccacaacggtcacaagtatgacgttcctgcattgaccaaactggcaaagttgcaattgaaccgagagttccaccttcctcgtgagaactgtattgacacccttgtgttgtc              <  1:285558‑M1/301‑282 (MQ=255)`  
                                                                                                                                                                                                                                                                                                             `ccATTATGatcgtttctgacatcgaagctaacgccctcttagagagcgtcactaagttccactgcggggttatctacgactactcaaccgctgagtacgtaagctaccgtccgagtgacttcggtgcgtatctggatgcgctggaagccgaggttgcacgaggcggtcttattgtgttccacaacggtcacaagtctgacgttcctgcattgaccaaactggcaaagttgcaattgaaccgagagttccaccttcctcgtgagaactgtattgacacccttgtgttgtcacgtttgattac  >  1:77719‑M1/1‑8 (MQ=255)`  

                                                                                                                                                                                                                                                                                                             `|`                                                                                                                                                                                                                                                                                                              
`ACTTTCCAGACGACTACGAGAAGTTTCGCTGGAGAGTCCCATTCTAATACGACTCACTAAAGGAGACACACCA‑TGTTCAAACTGATTAAGAAGTTAGGCCAACTGCTGGTTCGTATGTACAACGTGGAAGCCAAGCGACTGAACGATGAGGCTCGTAAAGAGGCCACACAGTCACGCGCTCTGGCGATTCGCTCCAACGAACTGGCTGACAGTGCATCCACTAAAGTTACCGAGGCTGCCCGTGTGGCAAACCAAGCTCAACAGCTTTCCAAATTCTTTGAGTAATCAAACA‑GGAGAAACCATTATGTCTAACGTAGCTGAAACTATCCGTCTATCCGATACAGCTGACCAGTGGAACCGTCGAGTCCACATCAACGTTCGCAACGGTAAGGCGACTATGGTTTACCGCTGGAAGGACTCTAAGTCCTCTAAGAATCACACTCAGCGTATGACGTTGACAGATGAGCAAGCACTGCGTCTGGTCAATGCGCTTACCAAAGCTGCCGTGACAGCAATTCATGAAGCTGGTCGCGTCAATGAAGCTATGGCTATCCTCGACAAGATTGATAACTAAGAGTGGTATCCTCAAGGTCGCCAAAG  >  NC_001604.1/13280‑13879`

  

Alignment Legend

Aligned base mismatch/match (shaded by quality score): `ATCG/ATCG` `< 3 ≤` `ATCG/ATCG` `< 9 ≤` `ATCG/ATCG` `< 20 ≤` `ATCG/ATCG` `< 34 ≤` `ATCG/ATCG` `< 38 ≤` `ATCG/ATCG`

Unaligned base: `atcg`    Masked matching base: `atcg`    Alignment gap: `‑`    Deleted base: `‑`
